# Identification of atypical hypoglycemia via continuous glucose monitoring in a patient presenting with hot flashes

**DOI:** 10.1002/jgf2.718

**Published:** 2024-07-10

**Authors:** Takuya Omura, Akemi Inami, Takahiro Kamihara, Yuki Tsuboi, Shuji Kawashima, Ken Tanaka, Taiki Sugimoto, Takashi Sakurai, Haruhiko Tokuda

**Affiliations:** ^1^ Department of Metabolic Research, Research Institute National Center for Geriatrics and Gerontology Obu Japan; ^2^ Department of Endocrinology and Metabolism, Hospital National Center for Geriatrics and Gerontology Obu Japan; ^3^ Department of Cardiology, Hospital National Center for Geriatrics and Gerontology Obu Japan; ^4^ Department of Family Medicine Harukaze Clinic Oita Japan; ^5^ Department of Public Health University of Hawaii at Manoa Honolulu Hawaii USA; ^6^ Department of Medicine University of Washington Seattle Washington USA; ^7^ Department of Prevention and Care Science, Research Institute National Center for Geriatrics and Gerontology Obu Japan; ^8^ Department of Clinical Laboratory, Hospital National Center for Geriatrics and Gerontology Obu Japan

## Abstract

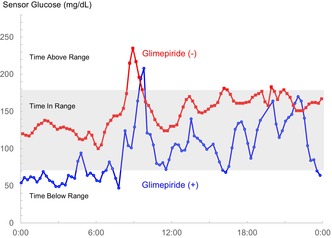

A 77‐year‐old female experienced persistent hot flashes for approximately several decades. No discernible abnormalities had been revealed by the endocrine screenings. She was referred to an endocrinologist because of the exacerbation of her nocturnal hot flashes and the emergence of hyperhidrosis. Her medical history is notable for type 2 diabetes mellitus (Category I) (Figure 1),^1^ hypertension, dyslipidemia, internal carotid artery stenosis, and uterine fibroids. Blood analyses revealed normal thyroid function, and estradiol levels below 24 pg/mL, LH at 16.48 mIU/mL, and FSH at 39.17 mIU/mL. The diminution of estradiol levels remained ambiguous; however, the LH and FSH concentrations were not elevated in the context of intact pituitary and adrenal function, thus making postmenopausal syndrome unlikely.

**FIGURE 1:**
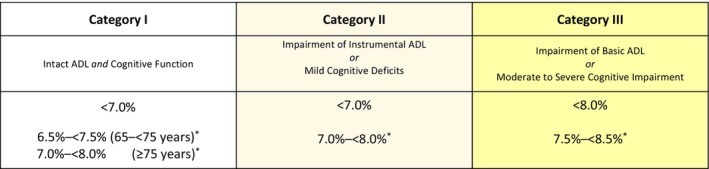
“Glycemic Targets for Elderly Patients with Diabetes” Outline. *The glycemic targets for individuals utilizing insulin formulations, sulfonylureas, glinides, etc., as delineated in the “Glycemic Targets for Elderly Patients with Diabetes” report by the Joint Committee of the Japan Diabetes Society and the Japan Geriatrics Society, are as follows. ADL, activities of daily living.

She was taking glimepiride, empagliflozin, vildagliptin, and metformin orally. Continuous glucose monitoring (CGM) was undertaken to explore the potential role of hypoglycemia in the genesis of her hot flashes, despite her HbA1c level of 7.1% being within the appropriate range (Figure 1). CGM disclosed periods of hypoglycemia, predominantly nocturnal, with glucose levels falling below 70 mg/dL (Figure 2). In response to these findings, the dosage of glimepiride was adjusted from 2 mg/day to 1 mg/day, resulting in the prompt relief of prolonged hot flashes and hyperhidrosis. Subsequently, glimepiride was discontinued (Figure 2).

**FIGURE 2:**
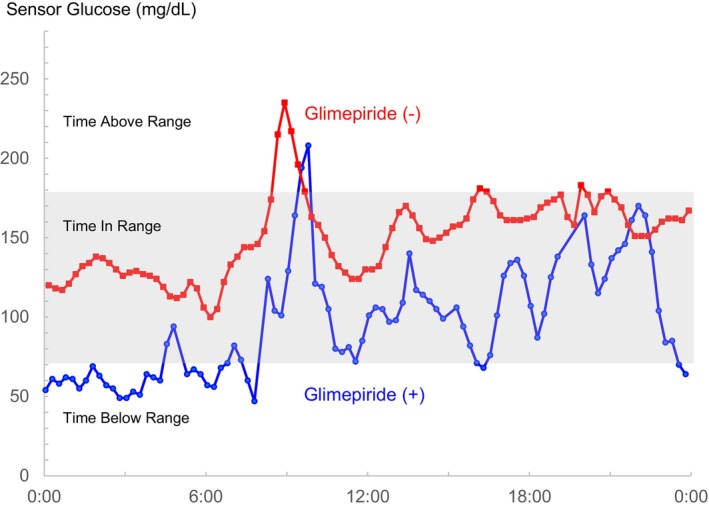
Persistent hot flashes: elucidating hypoglycemia through continuous glucose monitoring. The optimal sensor glucose level (time in range: 70–180 mg/dL) is depicted in grayscale, with the hypoglycemic range (time below range: <70 mg/dL) indicated below the grayscale area. The severity of hot flashes peaked at night. Continuous glucose monitoring (CGM) data during hypoglycemic episodes while the patient was on sulfonylurea medication (round) and following the discontinuation of sulfonylurea medication (square).

Symptoms associated with menopause are predominantly observed during the peri‐ and postmenopausal phases.^2^ In Japan, the median age at which women experience menopause is approximately 50 years,^3^ with the primary manifestation of menopausal symptoms occurring between the ages of 40 and 60. Consequently, the emergence of symptoms, such as hot flashes, in individuals over the age of 70, is unlikely to be attributed to menopausal disorders. Conversely, symptoms that appear in close proximity to the menopausal transition may persist into advanced age. Furthermore, it is posited that hypoglycemia may serve as a catalyst for hot flashes.^4^

In this case, the initial symptoms were ostensibly attributed to menopause, given the absence of hypoglycemic medication administration in her 40s. However, with the escalation of hypoglycemic medication, necessitated by the progressive worsening in glycemic control, the primary etiology of the patient's hot flashes likely transitioned to hypoglycemia. Although the exact onset of hypoglycemic episodes was unclear, this intensification may have precipitated a more pronounced susceptibility to hypoglycemia, particularly as hyperglycemia began to recede.

HbA1c is widely recognized as the gold standard for evaluating glycemic control. Nevertheless, it fails to capture the daily or circadian variations in blood glucose levels. Despite achieving optimal HbA1c targets, individuals may experience intermittent episodes of hypo‐ and hyperglycemia. Older patients, especially those with compromised renal function and on polypharmacy, are at an elevated risk for hypoglycemia events.^5^ In older patients, the symptoms of hypoglycemia are often unrecognized or varied, which may result in underlying and unexplained discomfort. CGM affords a graphical representation of glucose fluctuations over time, offering a potential explanation for nonspecific symptoms in the older population. However, it is essential to avoid any indiscriminate tapering of glycemic management.

## AUTHOR CONTRIBUTIONS

T.O. and H.T. conceptualized the study. T.O. and A.I. collected the clinical data. T.O., T.K., and H.T. drafted the manuscript. S.K. and K.T. was part of the preparations. Y.T., T.S. (Sugimoto), and T.S. (Sakurai) provided constructive comments on the draft. All authors have reviewed and approved the final version for publication.

## FUNDING INFORMATION

This study was supported by JSPS MEXT KAKENHI (JP23K16812) from the Japan Society for the Promotion of Science, Chukyo Medical Research Grant (2023.3.7.9) from Chukyo Longevity Medical Research and Promotion Foundation, and Research Funding for Longevity Science (21–1) from the National Center for Geriatrics and Gerontology. The funders played no role in the preparation of the manuscript.

## CONFLICT OF INTEREST STATEMENT

The authors have stated explicitly that there are no conflicts of interest in connection with this article.

## ETHICS APPROVAL STATEMENT

This study was approved by the local ethics committee of the National Centre for Geriatrics and Gerontology.

## INFORMED CONSENT STATEMENT

The patient has granted informed consent for the publication of this material, and comprehensive consent was obtained via website opt‐out. The information can be downloaded at: https://www.ncgg.go.jp/ncgg‐kenkyu/ekigaku/1724.html.

## Data Availability

Anonymized data will be available on request to any qualified investigator after approval by the Ethics Committee.
